# Elevated TYROBP expression predicts poor prognosis and high tumor immune infiltration in patients with low-grade glioma

**DOI:** 10.1186/s12885-021-08456-6

**Published:** 2021-06-23

**Authors:** Jiajie Lu, Yuecheng Peng, Rihong Huang, Zejia Feng, Yongyang Fan, Haojian Wang, Zhaorong Zeng, Yunxiang Ji, Yezhong Wang, Zhaotao Wang

**Affiliations:** Department of Neurosurgery, Institute of Neuroscience, the Second Affiliated Hospital of Guangzhou Medical University, Guangzhou, 510260 People’s Republic of China

**Keywords:** TYROBP, Prognosis, Immune infiltration, Low-grade glioma

## Abstract

**Background:**

Tyrosine protein tyrosine kinase binding protein (TYROBP) binds non-covalently to activated receptors on the surface of various immune cells, and mediates signal transduction and cellular activation. It is dysregulated in various malignancies, although little is known regarding its role in low-grade glioma. The aim of this study is to explore the clinicopathological significance, prognostic value and immune signature of TYROBP expression in low-grade glioma (LGG).

**Methods:**

The differentially expressed genes (DEGs) between glioma samples and normal tissues were identified from two GEO microarray datasets using the limma package. The DEGs overlapping across both datasets were functionally annotated by Gene Ontology (GO) and Kyoto Encyclopedia of Genes and Genomes (KEGG) analyses. STRING database was used to establish the protein-protein interaction (PPI) of the DEGs. The PPI network was visualized by Cytoscape and cytoHubba, and the core module and hub genes were identified. The expression profile of TYROBP and patient survival were validated in the Oncomine, GEPIA2 and CGGA databases. The correlation between TYROBP expression and the clinicopathologic characteristics were evaluated. Gene Set Enrichment Analysis (GSEA) and single-sample GSEA (ssGSEA) were performed by R based on the LGG data from TCGA. The TIMER2.0 database was used to determine the correlation between TYROBP expression and tumor immune infiltrating cells in the LGG patients. Univariate and multivariate Cox regression analyses were performed to determine the prognostic impact of clinicopathological factors via TCGA database.

**Results:**

Sixty-two overlapping DEGs were identified in the 2 datasets, and were mainly enriched in the response to wounding, focal adhesion, GTPase activity and Parkinson disease pathways. TYROBP was identified through the PPI network and cytoHubba. TYROBP expression levels were significantly higher in the LGG tissues compared to the normal tissues, and was associated with worse prognosis and poor clinicopathological parameters. In addition, GSEA showed that TYROBP was positively correlated to neutrophil chemotaxis, macrophage activation, chemokine signaling pathway, JAK-STAT signaling pathway, and negatively associated with gamma aminobutyric acid signaling pathway, neurotransmitter transport, neuroactive ligand receptor intersection etc. TIMER2.0 and ssGSEA showed that TYROBP expression was significantly associated with the infiltration of neutrophils, macrophages, myeloid dendritic cells and monocytes. The infiltration of the M2 phenotype macrophages, cancer-associated fibroblasts and myeloid dendritic cells correlated to worse prognosis in LGG patients. Finally, multivariate analysis showed that elevated TYROBP expression is an independent risk factor for LGG.

**Conclusion:**

TYROBP is dysregulated in LGG and correlates with immune infiltration. It is a potential therapeutic target and prognostic marker for LGG.

## Introduction

Gliomas are the most common primary tumors of the central nervous system, and are primarily composed of glial cells [1]. Diffuse low-grade and intermediate-grade gliomas (World Health Organization grades II and III), hereafter designated as lower-grade gliomas (LGGs), include astrocytomas, oligodendrogliomas and oligoastrocytomas [2, 3]. LGG patients survive longer compared to patients with higher-grade gliomas [4], with a survival period ranging from 1 to 15 years [5]. However, it is usually recalcitrant to conventional treatment, and may even progress to chronic impairment following radiotherapy [6]. Hence, it is crucial to identify novel prognostic markers of LGG in order to improve diagnosis and predict patient prognosis with greater accuracy.

Tyrosine protein tyrosine kinase binding protein (TYROBP) binds non-covalently to activated receptors on the surface of various immune cells, and mediates signal transduction and cellular activation [7–9]. Studies have demonstrated that DAP12 (also known as TYROBP) may play a dual role in the activation and inhibition of natural killer cells, myeloid cells, granulocytes, monocytes and other cells. When activated, it can activate natural killer cells and other immune inflammatory cells by activating PI3K-Akt, MAPK, PLCγ and its downstream signaling pathways. However, when inhibited, it may also lead to the inhibition of Toll-like receptor mediated activation, thereby inhibiting relevant inflammatory cells activation [10–12]. Besides, Qisheng Peng et al. showed that DAP12 inhibit LPS signaling in macrophages to prevent inflammation through physically combined with DOK3 [13]. TYROBP is an established oncogene for clear cell renal cell carcinoma and gastric cancer [14, 15]. Kopatz et al. showed that microglia can phagocytose glioma cells via the Siglec-h receptor for apoptosis induction, which indicates an important role of immune cell infiltration in glioma progression [16]. However, little is known regarding any potential biological function of TYROBP in glioma.

To this end, we first screened the differentially expressed genes (DEGs) between LGG and normal tissues from the GEO database, and identified TYROBP as a hub gene. TYROBP was significantly upregulated in the LGG patients compared to healthy controls, and predicted poorer survival outcome in the former. Gene set enrichment analysis (GSEA) based on The Cancer Genome Atlas (TCGA) indicated that TYROBP likely promotes LGG progression by regulating the tumor microenvironment. Consistent with this, ssGSEA and TIMER2.0 database further confirmed that the elevated TYROBP expression levels were associated with higher immune infiltration and poorer prognosis in LGG. Finally, TYROBP was identified as an independent risk factor for LGG. Our findings provide new insights into the oncogenic role of TYROBP in LGG, especially with regards to the immunological status of the tumor microenvironment.

## Method and materials

### Microarray data

**Fig. 1:**
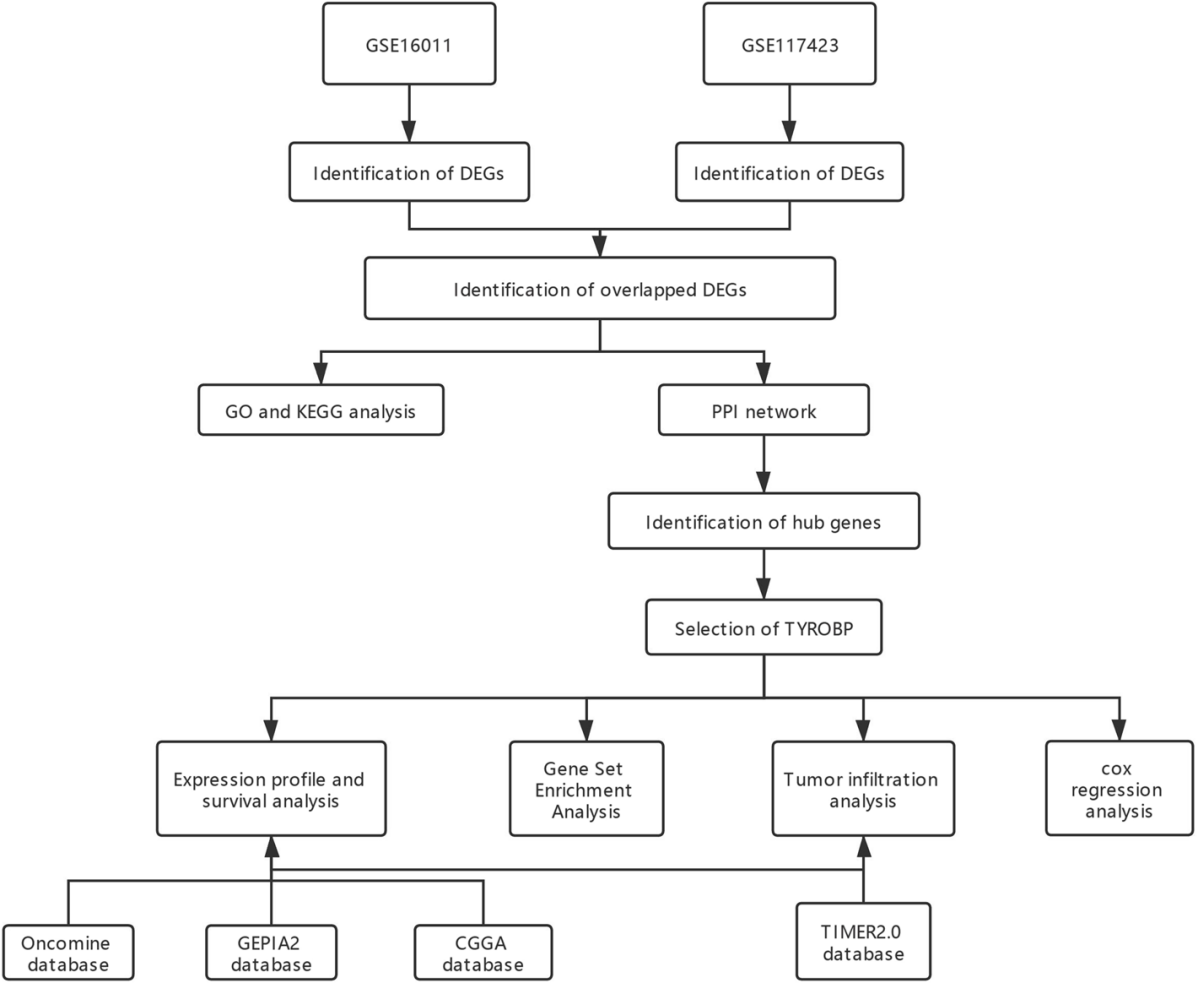
Flow diagram of study

The GSE16011 and GSE117423 datasets were downloaded from the NCBI Gene Expression Omnibus (GEO) database (https://www.ncbi.nlm.nih.gov/geo/) [17]. The GSE16011 array data of 276 glioma samples and 8 controls was submitted by Gravendeel et al., and GSE117423 array of 6 glioma tissues and 6 normal tissues was submitted by Vidyarthi et al. (Table 1). The flow chart of the study design is shown in Fig. 1.

**Table 1:** Details of GEO glioma data

GEO	Platform	Tumor	Normal	Total number of samples	Number of identified DEGs
GSE16011	GPL8542	276	8	284	2629
GSE16011	GPL16686	6	6	12	166

### Microarray data processing and DEGs screening

The GEO data was downloaded in the MINIML format. Following quality control, the raw data was standardized and converted to the log2 form. R Limma software (version: 3.4.2) was used to screen for the DGEs using |log fold change (FC)| ≥2 and *P* value < 0.05 as the thresholds in GSE16011 dataset, and |log FC| ≥ 1.5 and P value < 0.05 in the GSE117423 dataset in order to obtain a larger number of target genes. The Venn diagram of the DEGs from both datasets was drawn using R (version: 3.6.3) ggplot2 package, and the overlapping DEGs were identified.

### Functional enrichment analysis

Metascape (http://metascape.org) was used to functionally annotate the overlapping DEGs through the Custom Analysis module [18]. Significantly enriched gene Ontology (GO) terms (biological process, cellular component, and molecular function categories) and Kyoto Encyclopedia of Genes and Genomes (KEGG) pathways were identified using *P* value < 0.01, minimum overlap = 3 and enrichment factor > 1.5 as the criteria.

### Construction of protein-protein interaction (PPI) network and hub genes selection

The DEGs are imported into the STRING database (version: 11.0b, https://string-db.org) in order to identify the physically and functionally interacting genes and proteins [19]. The PPI network was constructed using combined score > 0.4 and further visualized by Cytoscape. The CytoHubba plug-in of Cytoscape version 3.8.2 (http://www.cytoscape.org/) [20, 21] was used to screen for the top 5 hub genes with the highest degree of interaction in the PPI network through the maximal clique centrality (MCC) algorithm.

### Expression analysis

The TYROBP mRNA expression profile was evaluated in Oncomine database [22] (https://www.oncomine.org). *P* value < 0.0001, fold change > 2.0, and genes ranking in the top 10% were set as thresholds. TYROBP mRNA expression levels in different subtypes of LGG were validated using data from GEPIA2 (http://gepia2.cancer-pku.cn/#index) [23] and CGGA (http://www.cgga.org.cn/). The data from Oncomine and CGGA [24, 25] were analyzed and visualized by R.

### Survival analysis

The LGG data from TCGA (https://portal.gdc.cancer.gov/) [26] and CGGA were incorporated in the Kaplan Meier survival analysis using the R survival and survminer packages. TIMER2.0 (http://timer.comp-genomics.org/) [27] databases was used to evaluate the effect of TYROBP expression in different LGG subtypes.

### Gene set enrichment analysis

Gene Set Enrichment Analysis (GSEA) is a computational method to determine whether a pre-defined set of genes shows significant differences between two biological states [28, 29]. To investigate the potential mechanisms underlying the impact of TYROBP expression on LGG progression, GSEA was conducted using R clusterProfiler package [30] to screen for biological pathways that showed significant differences between TYROBP^high^ and TYROBP^low^ groups. For each analysis, gene set permutations were implemented 5000 times. Gene sets with a false discovery rate (FDR) < 0.05 and adjusted *P* value < 0.05 were considered significantly enriched.

### Tumor infiltration analysis

The single-sample GSEA (ssGSEA) was performed using the R GSVA package [31] to quantify the tumor infiltration of 24 immune cell types based on TCGA . Feature gene panels for each immune cell type were obtained from a recent publication [32]. The TIMER2.0 database was then used to analyze the correlation between TYROBP expression and infiltration of neutrophils, macrophages, myeloid dendritic cells (DCs) and monocytes. *P* value < 0.01 was the threshold for significant association between TYROBP and immune cell infiltration. OS was analyzed as a function of TYROBP expression, M2 macrophage, myeloid dendritic cells (MDCs) and cancer-associated fibroblasts (CAFs).

### Univariate and multivariate cox regression analysis

Univariate and multivariate Cox analysis was used to determine the correlation of TYROBP expression and other clinicopathological factors (age, gender, race, grade, and radiation therapy) on OS, PFS and DSS with TCGA (https://portal.gdc.cancer.gov/) data. P value < 0.05 was set as the cut-off criterion. The P value, HR and 95% CI of each variable were calculated using the R forestplot package.

## Results

### Identification of DEGs

**Fig. 2:**
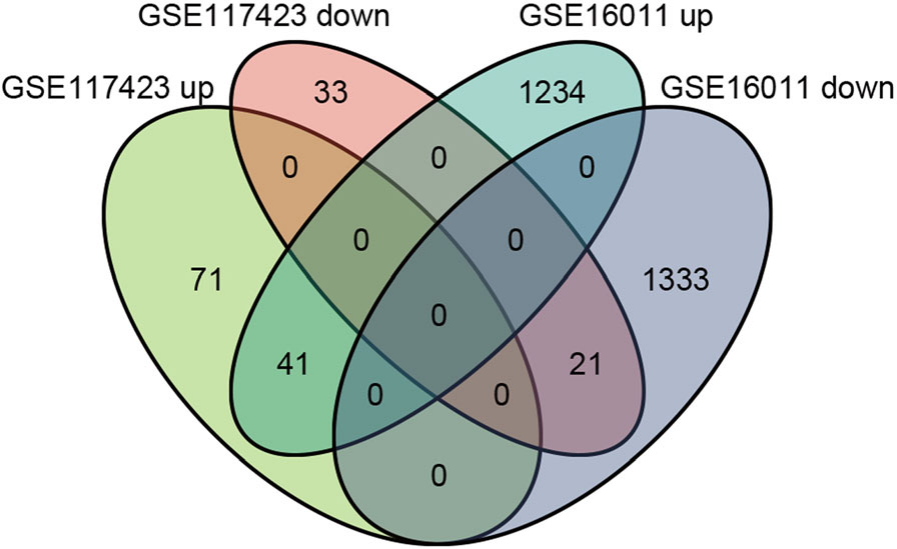
Venn diagram of DEGs. Comparison of two datasets with 2629 and 166 DEGs, revealing 41 up- and 21 down-regulated overlapping DEGs between glioma and normal tissues

A total of 2629 DEGs were identified in GSE16011 and 166 in GSE117423 after normalizing the chip results. As shown in Fig. 2, there were 62 overlapping DEGs between the two datasets, including 41 up- and 21 down-regulated genes.

### Functional annotation of TYROBP

**Fig. 3:**
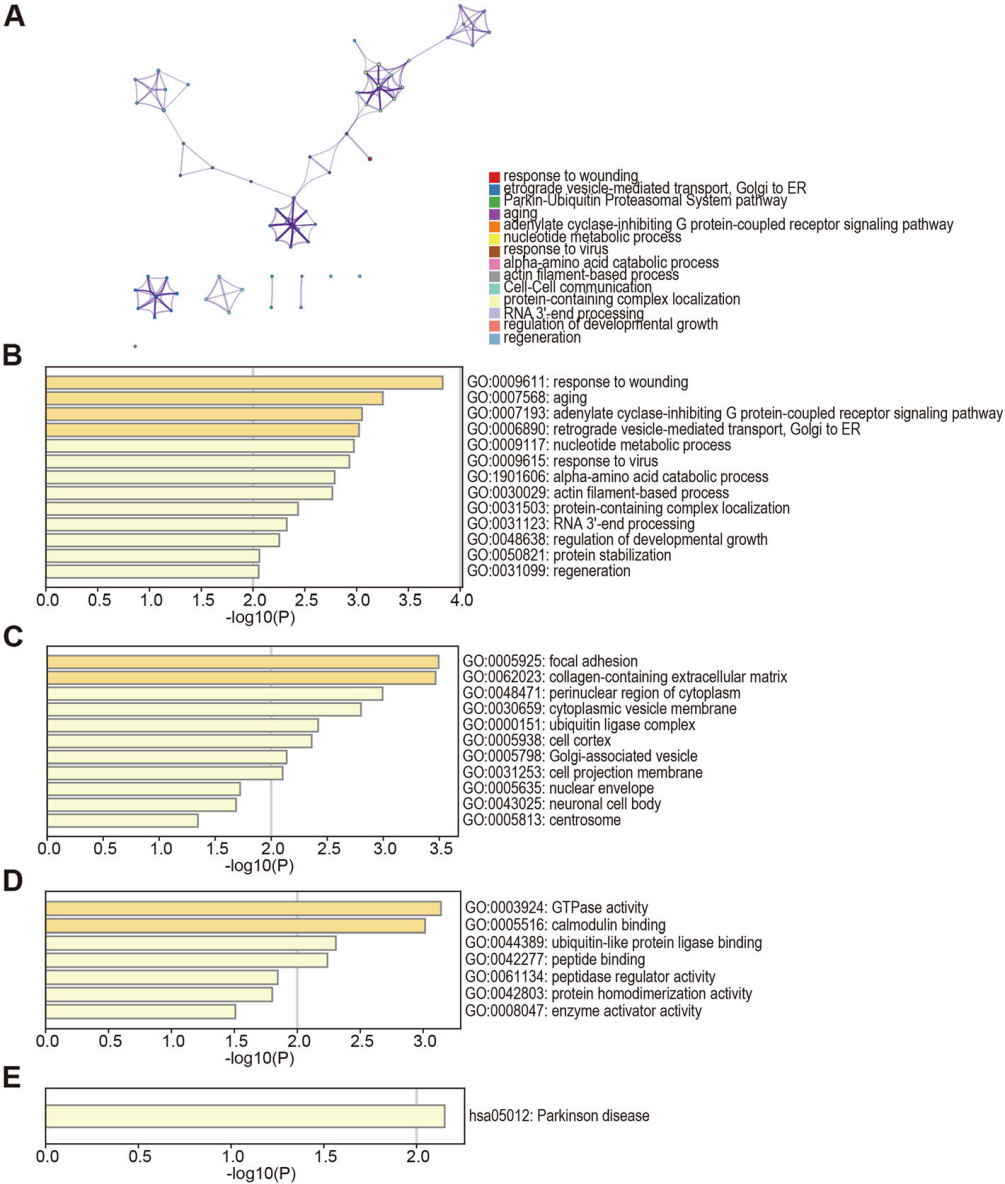
The enrichment analysis of 62 overlapping DEGs in glioma (Metascape). **(A)** An interactive network of the top 17 enriched terms color-coded by cluster-ID. Each color indicates one pathway. **(B-E)** Bar graph of GO terms, including BP, CC and MF, and KEGG pathways of the overlapping DEGs in glioma and normal tissues. The enriched terms are in orange. GO, Gene Ontologies; BP, Biological Process; CC, cellular component; MF, Molecular Feature; KEGG, Kyoto Encyclopedia of Genes and Genomes

The DEGs were functionally annotated using Metascape. As shown in Fig. 3A, with similarity > 0.3, the edges contained the links and each node indicated an enriched term represented by the cluster-ID in the network. The enriched terms consisted of response to wounding, Golgi-to-ER retrograde transport, Parkin-Ubiquitin Proteasomal System pathway etc. In addition, the most significantly enriched GO terms for biological processes (BP) were response to wounding, aging and adenylate cyclase-inhibiting G protein-coupled receptor signaling pathway, cellular component (CC) terms included focal adhesion, collagen-containing extracellular matrix and perinuclear region of cytoplasm, and the molecular functions (MF) terms were GTPase activity, calmodulin binding and ubiquitin-like protein ligase binding (Fig. 3B-D). The KEGG pathway related to Parkinson disease was also significantly enriched among the DEGs (Fig. 3E).

### PPI network construction and key gene selection

**Fig. 4:**
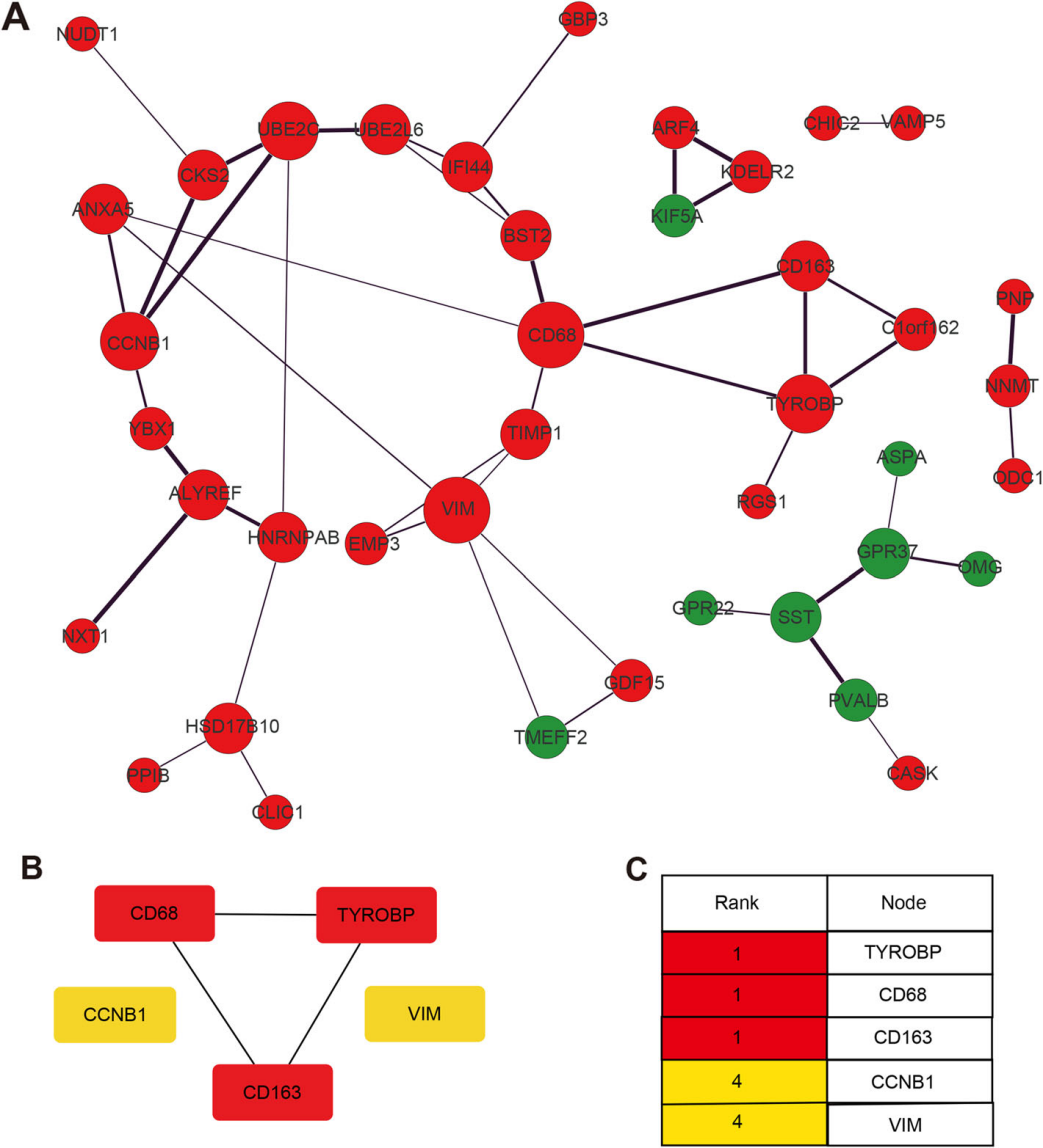
PPI network and hub genes identification. **(A)** The PPI network of DEGs was constructed by Cytoscape. Red nodes indicate up-regulated genes, green nodes indicate down-regulated genes, the node size is indicative of the degree of connectivity, and line thickness represents combined score. **(B-C)** The top five hub genes evaluated by cytoHubba and ranked by MCC. The intensity of red color indicated ranking. PPI, protein–protein interaction; MCC, maximal clique centrality

The PPI network of the overlapping DEGs was established by Cytoscape (Fig. 4A), and the first 5 hub genes identified via the CytoHubba plugin were ranked in terms of the MCC score. The most closely connected module was identified (Fig. 4B). Based on the rank and novelty, we select TYROBP as the key gene for further analysis (Fig. 4C).

### Prognostic significance of TYROBP

**Fig. 5:**
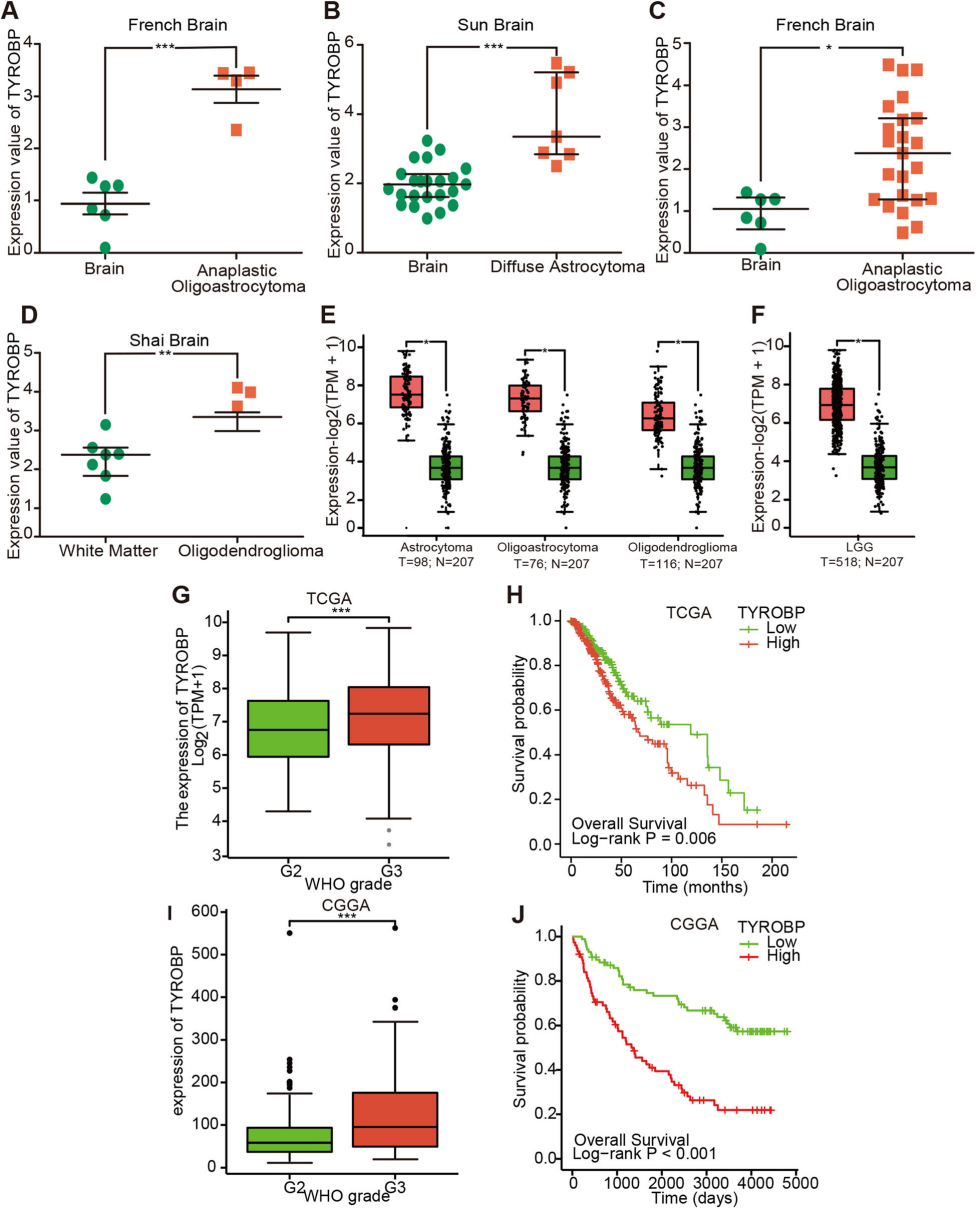
Differential TYROBP expression in LGG and correlation with survival. TYROBP expression in the different LGG subtypes and normal brain samples by Oncomine **(A-D)** and GEPIA2 **(E)**. The expression of TYROBP across LGG samples and normal tissues (GEPIA2) **(F)**. The relationship between tumor grade and TYROBP in LGG patients **(G, I)**. Kaplan-Meier curves of LGG patients stratified by TYROBP expression **(H, J)**. ns, *p* ≥ 0.05; *, *p* < 0.05; **, *p* < 0.01; ***, *p* < 0.001

Oncomine analysis of TYROBP in the LGG and normal tissues revealed that TYROBP was significantly up-regulated in the different LGG subtypes across multiple datasets (Fig. 5A-D). GEPIA2 analysis of 3 LGG subtypes and normal samples also indicated significantly higher levels of TYROBP in the LGG samples (Fig. 5E). The expression values of TYROBP in the LGG and normal tissues are summarized in Fig. 5F.

To determine the relationship between TYROBP and clinicopathological parameters in LGG patients, we analyzed the data from CGGA and TCGA databases. As shown in Fig. 5G and I, TYROBP expression was significantly associated with the tumor grade, and increased with advanced grade (*p* <  0.001). Elevated TYROBP in LGG was significantly associated with the WHO grade (G3 vs. G2, OR = 1.812, 95%CI [1.257–2.621], *P* = 0.002, IDH status (Mut vs. WT, OR = 0.357, 95%CI [0.219–0.568], *P* <  0 .001, 1p/19q codeletion (non-codel vs. codel, OR = 8.062, 95%CI [5.243–12.706], *P* <  0.001), P53 status (Mut vs. WT, OR = 3.23, 95%CI [2.24–4.67], *P* <  0 .001 and ATRX status (Mut vs. WT, OR = 3.21, 95%CI [2.17–4.75], *P* <  0 .001,while no significant correlation was seen with either gender or age (Table 2). Logistic regression analysis further indicated a correlation between TYROBP and adverse prognosis (Table 3). The influence of TYROBP expression on the survival of LGG patients was analyzed using TCGA portal and CGGA database. As shown in Fig. 5H and J, the survival of LGG patients with high TYROBP expression was significantly shorter (*p* = 0.006, *p* <  0.001). These results indicate that TYROBP functions as an oncogene in LGG, and its high expression level portend worse prognosis.

**Table 2:** Association of the expression profile of TYROBP mRNA with clinicopathological factors of low-grade glioma

Characteristic	Low expression of TYROBP	High expression of TYROBP	p
n	264	264	
WHO grade, n (%)			0.002
G2	128 (27.4%)	96 (20.6%)	
G3	103 (22.1%)	140 (30%)	
IDH status, n (%)			< 0.001
WT	29 (5.5%)	68 (13%)	
Mut	233 (44.4%)	195 (37.1%)	
1p/19q codeletion, n (%)			< 0.001
codel	139 (26.3%)	32 (6.1%)	
non-codel	125 (23.7%)	232 (43.9%)	
Age, meidan (IQR)	41 (33, 52.25)	39 (32, 53)	0.674
P53 status, n (%)			< 0.001
WT	172 (42.79%)	101 (25.12%)	
Mut	79 (19.65%)	150 (37.31%)	
ATRX status, n (%)			< 0.001
WT	198 (39.44%)	135 (26.89%)	
Mut	53 (10.56%)	116 (23.11%)

**Table 3:** TYROBP expression correlated with clinicopathological characteristics

Characteristics	Total(N)	Odds Ratio(OR)	P value
Gender (Female vs. Male)	528	0.898 (0.637–1.266)	0.541
Age (> 40 vs. <=40)	528	0.886 (0.629–1.246)	0.486
WHO grade (G3 vs. G2)	467	1.812 (1.257–2.621)	0.002
IDH status (Mut vs. WT)	525	0.357 (0.219–0.568)	< 0.001
1p/19q codeletion (non-codel vs. codel)	528	8.062 (5.243–12.706)	< 0.001

### Putative functional role of TYROBP in LGG

**Fig. 6:**
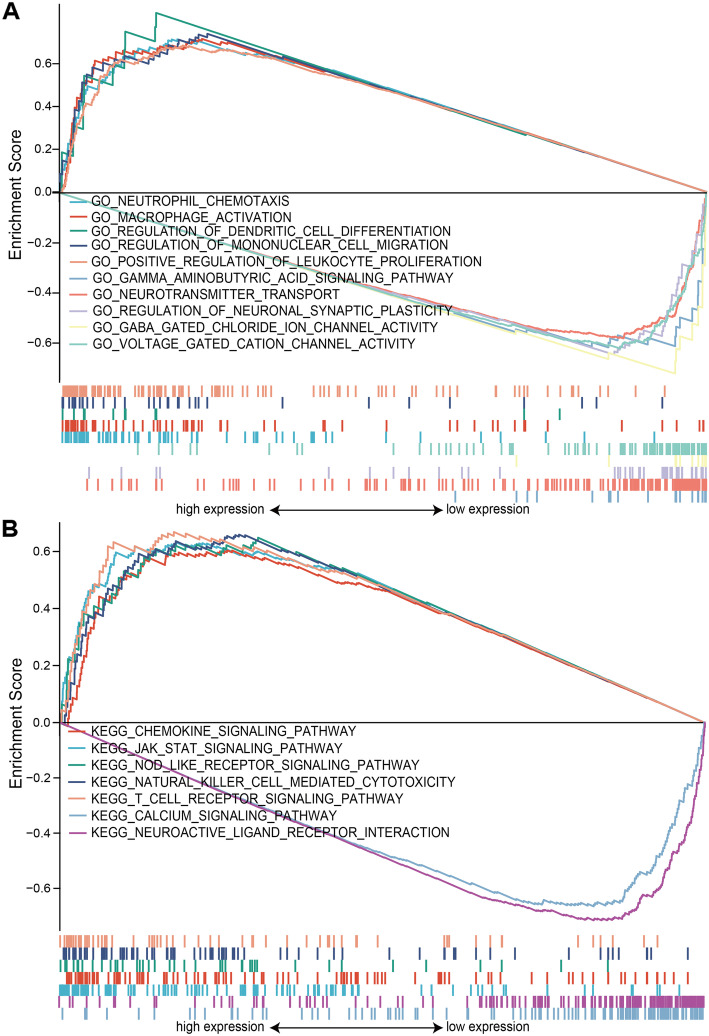
Functional annotation of TYROBP in LGG. **(A)** GSEA results showing differential enrichment of GO terms as a function of TYROBP expression. Top 5 GO terms for TYROBP^high^ - neutrophil chemotaxis, macrophage activation, regulation of dendritic cell differentiation, regulation of mononuclear cell migration and positive regulation of leukocyte proliferation. Top 5 GO terms for TYROBP^low^ - gamma aminobutyric acid signaling pathway, neurotransmitter transport, regulation of neuronal synaptic plasticity, GABA gated chloride ion channel activity and voltage gated cation channel activity. **(B)** GSEA results showing differential enrichment of KEGG pathways as a function of TYROBP. Top 5 KEGG pathways for TYROBP^high^ - chemokine signaling pathway, JAK-STAT signaling pathway, NOD like receptor signaling pathway, natural killer cell mediated cytotoxicity and T cell receptor signaling pathway. Two KEGG pathways in TYROBP^low^ - calcium signaling pathway and neuroactive ligand receptor intersection. All results of GSEA were based on NES, adjusted *P* value and FDR value. GSEA, gene set enrichment analysis

GSEA was used to distinguish between TYROBP^high^ and TYROBP^low^ LGG in terms of GO and KEGG enrichment (adjust *P* value < 0.05, FDR <  0.05). Neutrophil chemotaxis, macrophage activation, regulation of dendritic cell differentiation, regulation of mononuclear cell migration and positive regulation of leukocyte proliferation were the significantly enriched GO terms in the TYROBP^high^ phenotype, whereas gamma aminobutyric acid signaling pathway, neurotransmitter transport, regulation of neuronal synaptic plasticity, GABA gated chloride ion channel activity and voltage gated cation channel activity were significantly enriched in the TYROBP^low^ phenotype (Fig. 6A). The top 5 enriched KEGG pathways in TYROBP^high^ LGG were the chemokine signaling, JAK-STAT, NOD-like receptor, natural killer cell-mediated cytotoxicity and T cell receptor signaling pathways. In contrast, only the calcium signaling pathway and neuroactive ligand receptor intersection were significantly enriched in the TYROBP^low^ phenotype (Fig. 6B). The GO and KEGG items are summarized in Table 4. Thus, TYROBP is involved in LGG development and progression.

**Table 4:** Gene enrichment analysis based on high and low TYROBP expression phenotype

Gene set name	NES	P.adjust	FDR
high expression			
GO_NEUTROPHIL_CHEMOTAXIS	1.546	0.003	0.002
GO_MACROPHAGE_ACTIVATION	1.547	0.003	0.002
GO_REGULATION_OF_DENDRITIC_CELL_DIFFERENTIATION	1.595	0.012	0.009
GO_REGULATION_OF_MONONUCLEAR_CELL_MIGRATION	1.542	0.003	0.002
GO_POSITIVE_REGULATION_OF_LEUKOCYTE_PROLIFERATION	1.507	0.003	0.002
KEGG_CHEMOKINE_SIGNALING_PATHWAY	1.394	0.004	0.004
KEGG_JAK_STAT_SIGNALING_PATHWAY	1.328	0.007	0.006
KEGG_NOD_LIKE_RECEPTOR_SIGNALING_PATHWAY	1.369	0.038	0.033
KEGG_NATURAL_KILLER_CELL_MEDIATED_CYTOTOXICITY	1.435	0.004	0.004
KEGG_T_CELL_RECEPTOR_SIGNALING_PATHWAY	1.442	0.004	0.004
low expression			
GO_GAMMA_AMINOBUTYRIC_ACID_SIGNALING_PATHWAY	−1.546	0.013	0.011
GO_NEUROTRANSMITTER_TRANSPORT	−1.611	0.004	0.004
GO_REGULATION_OF_NEURONAL_SYNAPTIC_PLASTICITY	−1.688	0.004	0.004
GO_GABA_GATED_CHLORIDE_ION_CHANNEL_ACTIVITY	−1.735	0.004	0.004
GO_VOLTAGE_GATED_CATION_CHANNEL_ACTIVITY	−1.698	0.004	0.004
KEGG_CALCIUM_SIGNALING_PATHWAY	−1.509	0.009	0.009
KEGG_NEUROACTIVE_LIGAND_RECEPTOR_INTERACTION	−1.644	0.009	0.009

NES: normalized enrichment score; P.adjust: adjust P value; FDR: false discovery rate

Gene sets with adjust *p*-value < 0.05 and FDR q-value < 0.05 are considered as significant

### Tumor infiltration analysis

**Fig. 7:**
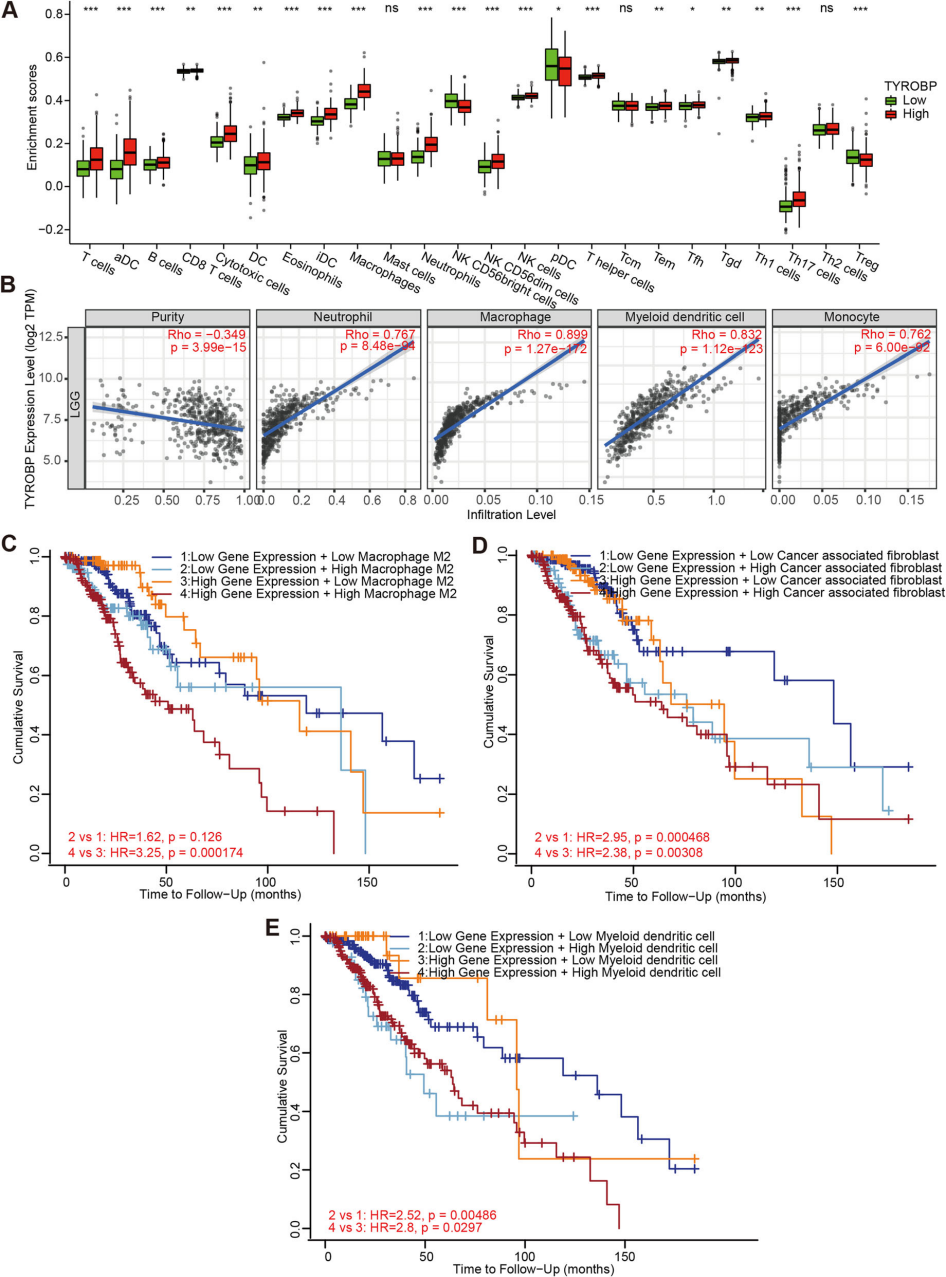
Correlation between immune infiltrates and TYROBP expression in LGG. Correlation between TYROBP expression and 24 tumor-infiltrating immune cell types **(A)**. TYROBP expression was positively correlated with neutrophil, macrophage, myeloid dendritic cell and monocyte **(B)**. Higher infiltration of macrophage M2 **(C)**, cancer associated fibroblast **(D)** and myeloid dendritic cell **(E)** correlated with worse prognosis. P value < 0.05 was considered statistically significant. ns, *p* ≥ 0.05; *, *p* < 0.05; **, *p* < 0.01; ***, *p* < 0.001

The extent of lymphocyte infiltration in tumor tissues is an independent prognostic factor of survival and sentinel lymph node status in various neoplasms. Therefore, we analyzed the correlation between TYROBP expression and infiltrating immune cells in LGG. As shown in Fig. 7A, TYROBP expression correlated significantly with the infiltration of T cells, activated DCs (aDCs), B cells, cytotoxic cells, eosinophils, immature DCs (iDCs), macrophages, neutrophils, CD56^bright^ NK cells, CD56^dim^ NK cells, NK cells, T helper cells, Th17 cells, Treg (*P* <  0.001), CD8 T cells, DCs, Tgd, Th1 cells, T effector memory (Tem) cells (*P* < 0.01), plasmacytoid DCs (pDCs) and T follicular helper (Tfh) cells (*P* < 0.05). In contrast, no significant association was found between TYROBP expression and mast cells, T central memory (Tcm) cells and Th2 cells infiltration (Fig. 7A). Furthermore, TIMER2.0 showed that the expression of TYROBP also significantly correlated with the infiltration of neutrophils (r = 0.767, *P* = 8.48e-94), macrophages (r = 0.899, *P* = 1.27e-172), myeloid DCs (r = 0.832, *P* = 1.12e-123) and monocytes (r = 0.762, *P* = 6.00e-92) in LGG (Fig. 7B). Finally, higher M2 macrophage infiltration was associated with poor prognosis for TYROBP^high^ LGG (Fig. 7C; HR = 3.25, *p* = 0.000174). Similarly, higher CAF and myeloid DC infiltration also correlated with worse outcome in LGG (Fig. 7D-E).

### Cox regression analysis

**Fig. 8:**
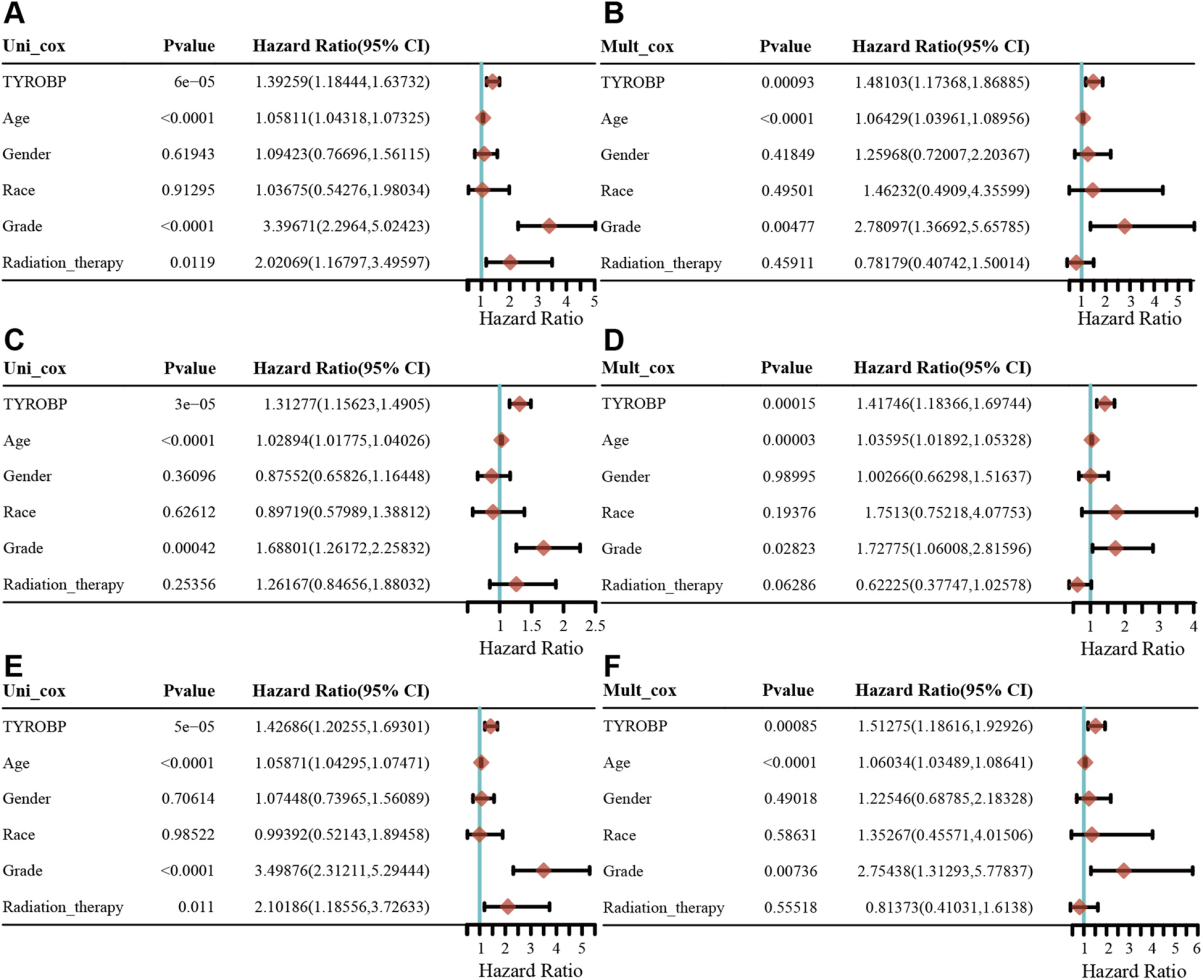
Univariate and Multivariate Cox analysis of TYROBP expression and other clinical pathological factors for OS (**A-B**), PFS (**C-D**) and DSS (**E-F**). (Covariates -TYROBP expression, age, gender, race, grade and radiation therapy independent prognostic factors). OS, overall survival; PFS, progression free survival; DSS, disease specific survival; HR, hazard ratio

Univariate Cox regression analysis showed that TYROBP was significantly associated with the OS (HR 1.39259, 95% CI = 1.18444,1.63732, *p* = 6e-05), PFS (HR 1.31277, 95% CI = 1.15623,1.44925, *p* = 3e-05) and DSS (HR 1.42686, 95% CI = 1.20255,1.6930, *p* = 5e-05). In addition, the multivariate Cox regression analysis showed that TYROBP was independent risk factor for OS (HR 1.48103, 95% CI = 1.17368, 1.86885, *p* = 0.00093), PFS (HR 1.18366, 1.41746, 95% CI =1.69744, *p* = 0.00015) and DSS (HR 1.51275, 95% CI = 1.18616,1.92926, *p* = 0.00052). The results are summarized in Fig. 8.

## Discussion

Glioma is an aggressive malignancy of the central nervous system, and shows rapid recurrence after standardized temozolomide chemotherapy and radiotherapy [33]. Currently, gliomas are classified into the high-grade and low grade tumors, and the survival of LGG patients ranges from 1 to 15 years [34]. LGG tumors cannot be completely excised due to their invasive nature, and progress frequently to glioblastoma [35]. Therefore, the clinical prognosis of LGG would greatly improve if diagnosed at the early stage. Bioinformatics is a high-throughput approach that can quickly and accurately identify biomarkers associated with the development of LGG. In the present study, we found that TYROBP was significantly upregulated in the LGG tissues compared to normal samples, and its high expression levels correlated with adverse clinicopathological parameters and worse prognosis, which indicated that we could determine the WHO grade, IDH status, and 1p/19q codeletion to a certain extent through the detection of TYROBP, which makes it possible for us to carry out clinical work in the future and provide precise and individualized treatment for patients. In addition, TYROBP overexpression was established as an independent risk factor in LGG patients. The, TYROBP is a potential prognostic factor in patients with LGG.

Infiltration of immune cells into solid tumors is a significant factor influencing tumor genesis and progression. For instance, DAP12 mediates acute non-infectious lung tissue injury by activating the tissue-resident alveolar macrophages, and increasing neutrophil infiltration [36]. In addition, Siglecc-15 promotes TGF-β secretion by tumor-associated macrophages via the DAP12-SYK pathway, which in turn accelerates neoplasm progression [37]. TIMER2.0 database analysis confirmed that higher TYROBP expression correlated to increased infiltration of neutrophils, macrophages, myeloid DCs and monocytes, suggesting that TYROBP may negatively impact LGG prognosis by regulating the immune microenvironment. However, the underlying mechanisms have to be validated further. To this end, we functionally annotated TYROBP through GO terms and KEGG pathways, and found that TYROBP overexpression positively correlated with neutrophil chemotaxis, macrophage activation, regulation of DC differentiation, regulation of mononuclear cell migration and positive regulation of leukocyte proliferation, thus underscoring the immunoregulatory role of TYROBP in LGG. Moreover, high expression levels of TYROBP negatively correlated with gamma aminobutyric acid signaling pathway, neurotransmitter transport, regulation of neuronal synaptic plasticity, GABA gated chloride ion channel activity, and voltage gated cation channel activity. The GABA receptor has been detected in low-level glioma cells and cell lines [38–40], and its down-regulation leads to uncontrolled proliferation and progression of glioblastoma [40, 41]. Therefore, TYROBP may regulate LGG by targeting GABA receptors. High TYROBP expression was positively correlated with chemokine signaling pathway, JAK-STAT signaling pathway, NOD like receptor signaling pathway, natural killer cell mediated cytotoxicity, and T cell receptor signaling pathway. Studies show that the JAK/STAT and NOD like receptor signal pathways are activated during the malignant progression of gliomas [42, 43]. Taken together, elevated TYROBP in LGG may lead to poor prognosis by increasing immune cell infiltration in the tumor microenvironment through the activation of these pathways. However, more studies are required to determine the potential mechanisms.

TYROBP, also known as KARAP/DAP12 (12 kDa Killer Cell Activated Receptor-related protein/DNAX activating protein), is primarily expressed in myeloid cells and natural killer cells and stimulate various signaling pathways upon binding to immune receptors [44]. Studies show that high TYROBP expression in breast cancer cells is correlated with bone metastasis and poor prognosis [45]. Besides, Ping Wu et al. and Junjie Jiang et al. reveal that TYROBP is a potential prognostic biomarker for clear cell renal cell carcinoma and gastric cancer [14, 15]. Although the correlation between TYROBP expression and LGG has not been elucidated so far, our results indicate that TYROBP is a prognostic biomarker of LGG plays an essential role in its progression.

This study has some limitations that ought to be considered. Due to technical limitations, we did not verify the in-silico data on clinical samples. In addition, the mechanistic role of TYROBP in LGG genesis and progression have to be validated by in vitro and in vivo functional studies. Nevertheless, we established TYROBP overexpression as an independent factor of poor prognosis in LGG, which provides new insights into the pathological mechanisms underlying LGG progression, especially in the context of the tumor immunological environment.

## Data Availability

The datasets and materials of this study are derived from public databases, as follows. NCBI Gene Expression Omnibus (GEO) database (https://www.ncbi.nlm.nih.gov/geo/), Metascape database(http://metascape.org), STRING database (https://string-db.org), Cytoscape software (http://www.cytoscape.org/), Oncomine database (https://www.oncomine.org), Gene Expression Profiling Interactive analysis 2 (GEPIA2) database (http://gepia2.cancer-pku.cn/#index), Chinese Glioma Genome Atlas (CGGA) database (http://www.cgga.org.cn/), The Cancer Genome Atlas (TCGA) database (https://portal.gdc.cancer.gov/), TIMER2.0 database (http://timer.comp-genomics.org/).
